# Supplementary material for: Optimal density of bacterial cells
Source: PLoS Comput Biol. 2023 Jun 12;19(6):e1011177. doi: 10.1371/journal.pcbi.1011177 (PMC10289677; doi:10.1371/journal.pcbi.1011177)
Supplement: S1 Fig — Metabolic reactions (blue curves), which are assumed to be saturated by the substrates, do not display any optimal cytosolic density at any intermediate values. In contrast, ribosomal reactions (red curves), which are assumed to be in the diffusion limit, show optimal densities. Solid lines are for smaller systems with N = 20 reactions, dashed lines are for larger systems of N = 100 reactions. (DOCX) [file pcbi.1011177.s001.docx]

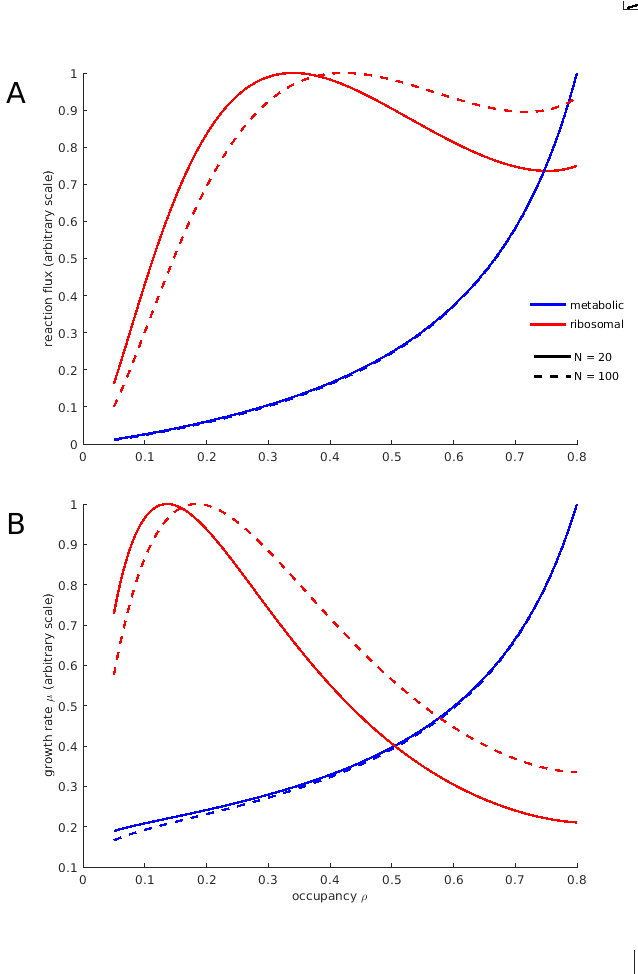


**Supplementary Figure S1.** **(A) reaction fluxes and (B) growth rate (flux per unit dry mass) of the linear model based on the treatment of crowding effects by Vazquez [1].** Metabolic reactions (blue curves), which are assumed to be saturated by the substrates, do not display any optimal cytosolic density at any intermediate values. In contrast, ribosomal reactions (red curves), which are assumed to be in the diffusion limit, show optimal densities. Solid lines are for smaller systems with *N*=20 reactions, dashed lines are for larger systems of *N*=100 reactions.

# References

1. Vazquez, A. (2010). Optimal cytoplasmatic density and flux balance model under macromolecular crowding effects. In Journal of Theoretical Biology (Vol. 264, Issue 2, pp. 356–359). Elsevier BV. <https://doi.org/10.1016/j.jtbi.2010.02.024>
