# Supplementary material for: Optimal density of bacterial cells
Source: PLoS Comput Biol. 2023 Jun 12;19(6):e1011177. doi: 10.1371/journal.pcbi.1011177 (PMC10289677; doi:10.1371/journal.pcbi.1011177)
Supplement: S2 Fig — This applies to a modified whole cell model with more diffusion efficient metabolic reactions (θ = 4.6 for metabolic reactions and θ = 2.3 for ribosomal reactions; thick solid curves) compared with the main model (θ = 2.3 for both types of reactions; thin broken curves; replicates of Fig 5). (DOCX) [file pcbi.1011177.s002.docx]

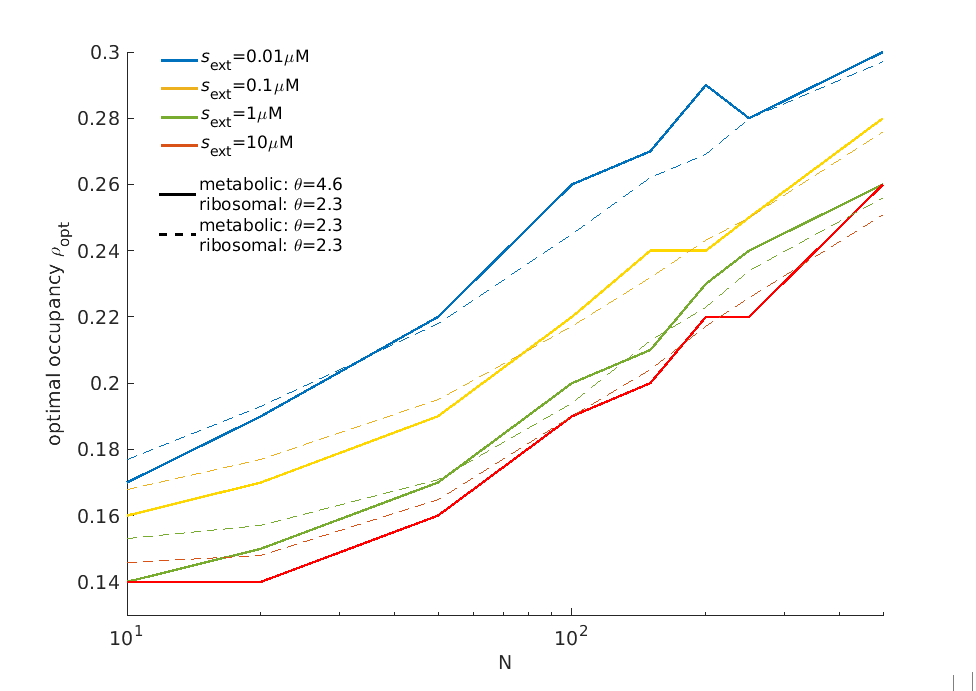


**Supplementary Figure S2. The optimal cytosolic occupancy increases with metabolic pathway length *N* and decreasing external nutrient concentration *s*_ext_.** This applies to a modified whole cell model with more diffusion efficient metabolic reactions (*θ*=4.6 for metabolic reactions and *θ*=2.3 for ribosomal reactions; thick solid curves) compared with the main model (*θ*=2.3 for both types of reactions; thin broken curves; replicates of Fig. 5).
