# Supplementary material for: Optimal density of bacterial cells
Source: PLoS Comput Biol. 2023 Jun 12;19(6):e1011177. doi: 10.1371/journal.pcbi.1011177 (PMC10289677; doi:10.1371/journal.pcbi.1011177)
Supplement: S3 Fig — Each column of plots shows data for a different nutrient concentration in the environment, sext (in μM). (DOCX) [file pcbi.1011177.s003.docx]

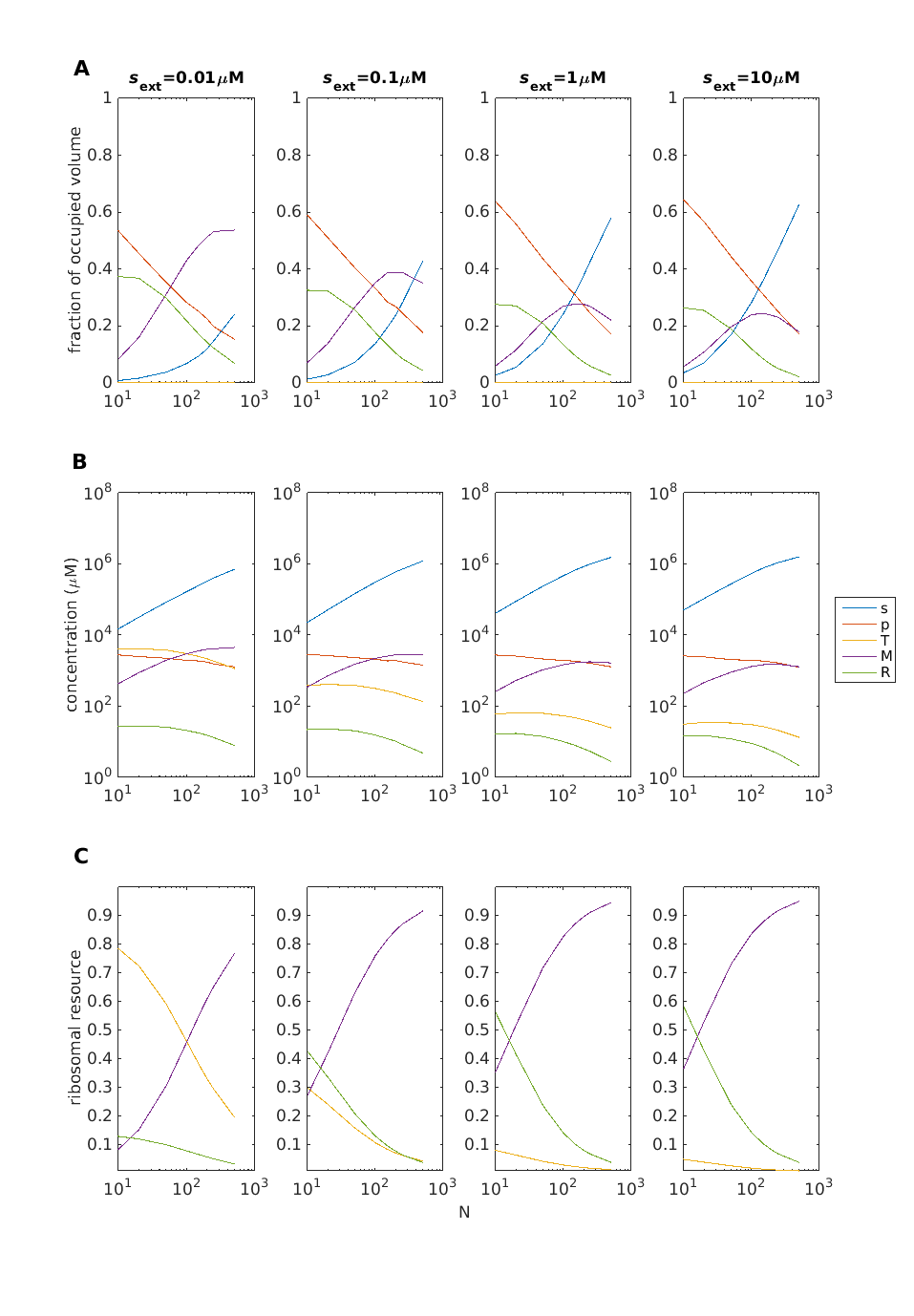


**Supplementary Figure S3.** The dependence on the number of active metabolic reactions *N* for the volume fraction **(A)** and concentration **(B)** of substrate *s*, protein precursor *p*, transporter *T*, metabolic enzyme *M*, and ribosome *R*, and the proportion of ribosomal activities dedicated to the three types of proteins **(C)**. Each column of plots shows data for a different nutrient concentration in the environment, *s*_ext_ (in µM).
