# Supplementary material for: Optimal density of bacterial cells
Source: PLoS Comput Biol. 2023 Jun 12;19(6):e1011177. doi: 10.1371/journal.pcbi.1011177 (PMC10289677; doi:10.1371/journal.pcbi.1011177)
Supplement: S4 Fig — Each column of plots shows data for a different nutrient concentration in the environment, sext (in μM). (DOCX) [file pcbi.1011177.s004.docx]

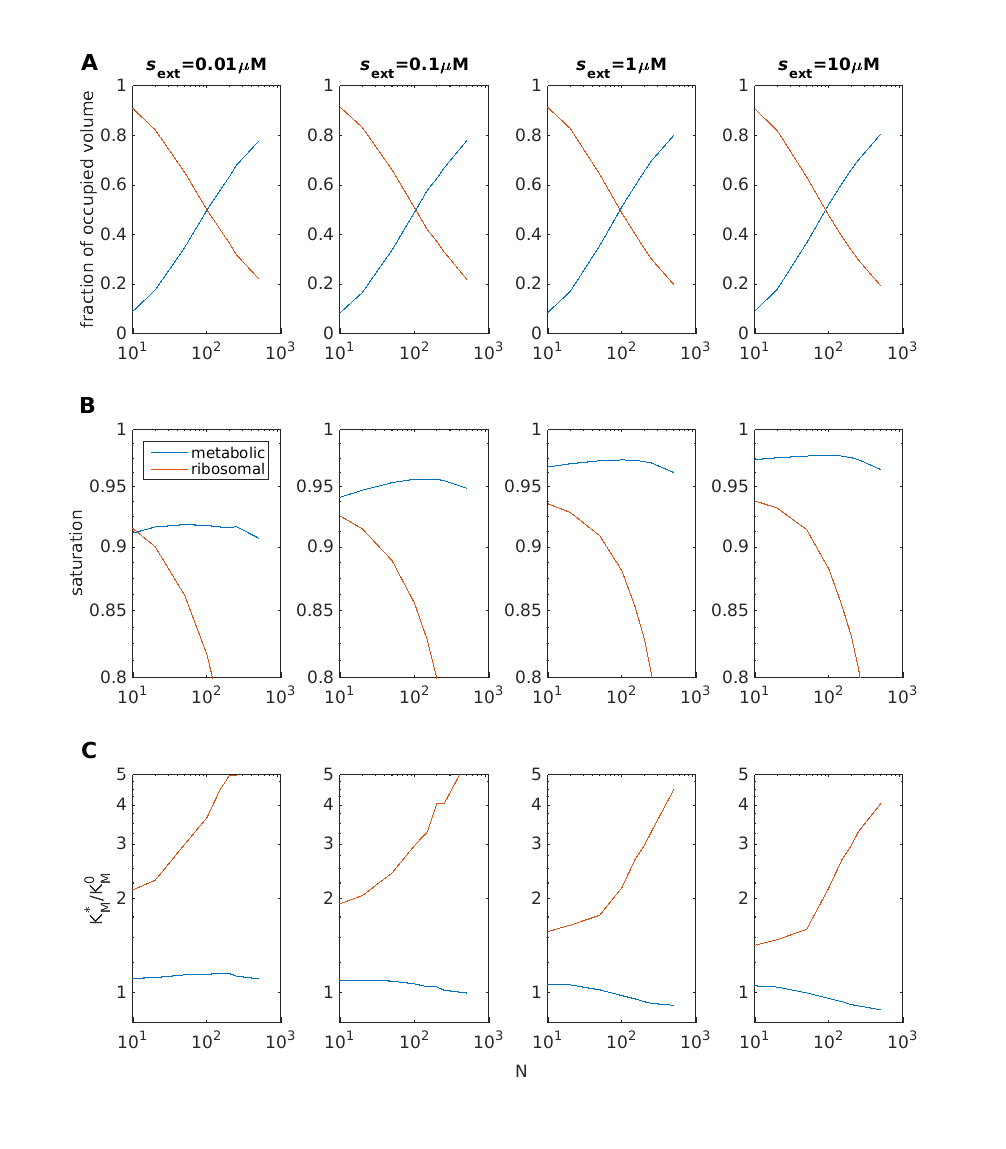


**Supplementary Figure S4.** The volume fraction **(A)**, substrate saturation **(B)**, and

$$K_{\text{M}}^{*}$$

 **(C)** of the metabolic sector (blue) and ribosomal sector (red), plotted against *N*. Each column of plots shows data for a different nutrient concentration in the environment, *s*_ext_ (in µM).
