# Supplementary material for: Optimal density of bacterial cells
Source: PLoS Comput Biol. 2023 Jun 12;19(6):e1011177. doi: 10.1371/journal.pcbi.1011177 (PMC10289677; doi:10.1371/journal.pcbi.1011177)
Supplement: S5 Fig — (DOCX) [file pcbi.1011177.s005.docx]

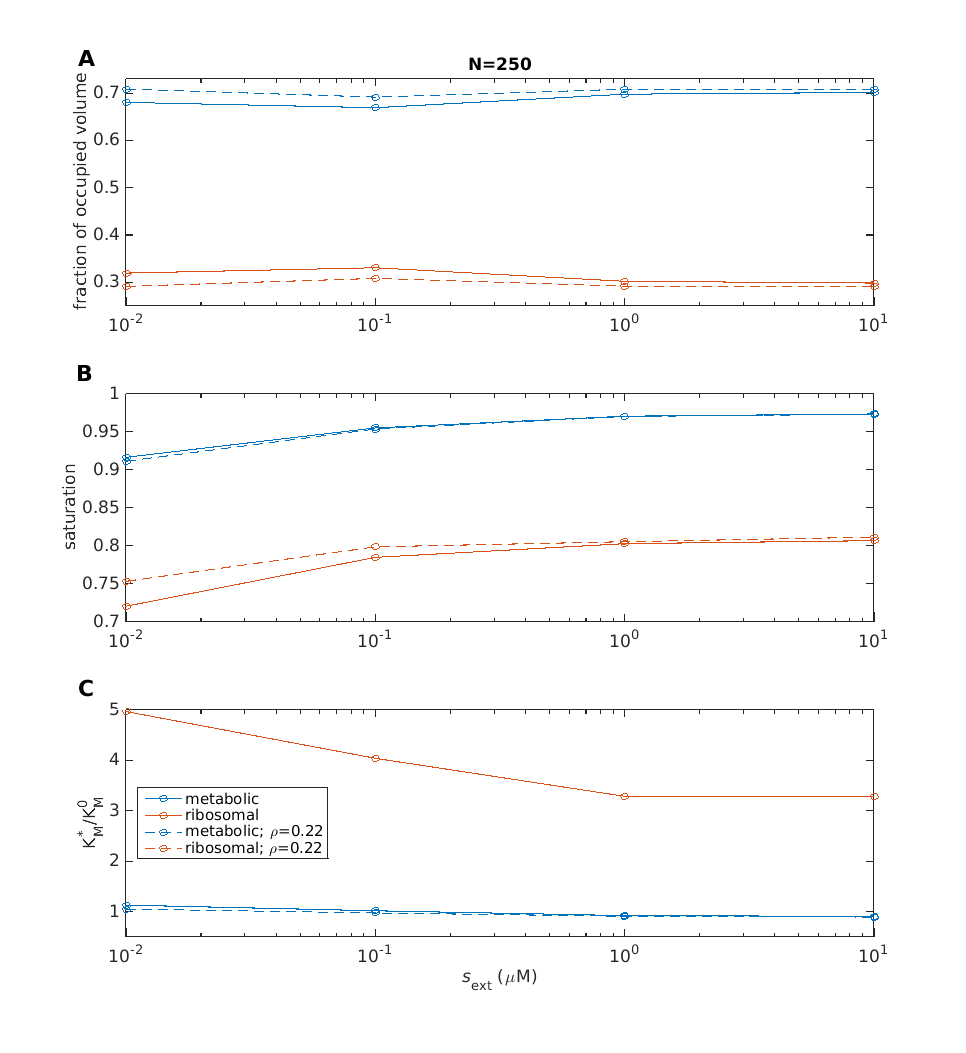


**Supplementary Figure S5.** The volume fraction **(A),** saturation **(B)** and crowding-adjusted Michaelis parameter $K_{\text{M}}^{*}$ **(C)** of the metabolic sector (blue) and ribosomal sector (red), plotted against different nutrient concentrations in the environment, with fixed pathway length *N*=250, at optimal occupancy (solid curves), and with fixed occupancy *ρ*=0.22 (broken curves).
