# Supplementary material for: Optimal density of bacterial cells
Source: PLoS Comput Biol. 2023 Jun 12;19(6):e1011177. doi: 10.1371/journal.pcbi.1011177 (PMC10289677; doi:10.1371/journal.pcbi.1011177)
Supplement: S6 Fig — (DOCX) [file pcbi.1011177.s006.docx]

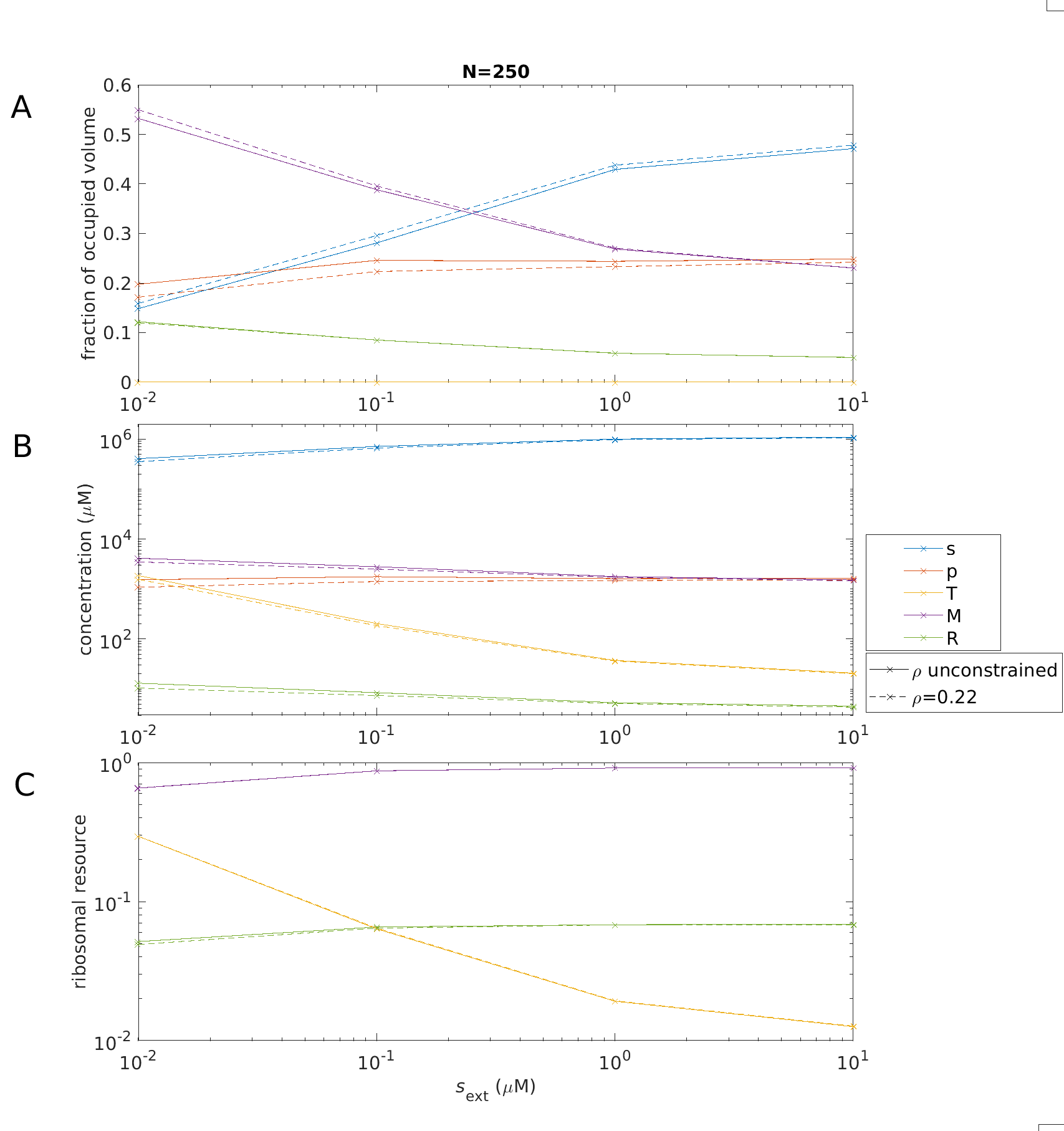


**Supplementary Figure S6.** The volume fraction **(A)** and concentration **(B)** of substrates *s*, precursor *p*, transporter *T*, metabolic enzymes *M*, and ribosome *R*, and also the proportion of ribosomal activities dedicated to the three types of proteins **(C)**, plotted against different nutrient concentrations in the environment, with *N* fixed at 250 (solid curves), and with *N* fixed at 250 and *ρ* fixed at 0.22 (dashed curves).
