# Supplementary material for: Optimal density of bacterial cells
Source: PLoS Comput Biol. 2023 Jun 12;19(6):e1011177. doi: 10.1371/journal.pcbi.1011177 (PMC10289677; doi:10.1371/journal.pcbi.1011177)
Supplement: S7 Fig — Simulations considered the individual and combined effects of diffusion limitation and transition state limitation for a parallel system of ribosomal reactions (red) and for a linear system of metabolic reactions (blue: θ = 2.3; green: combined effect with a stronger bias towards transition state limitation, θ = 4.6). (A) N = 20 and (B) N = 100. (DOCX) [file pcbi.1011177.s007.docx]

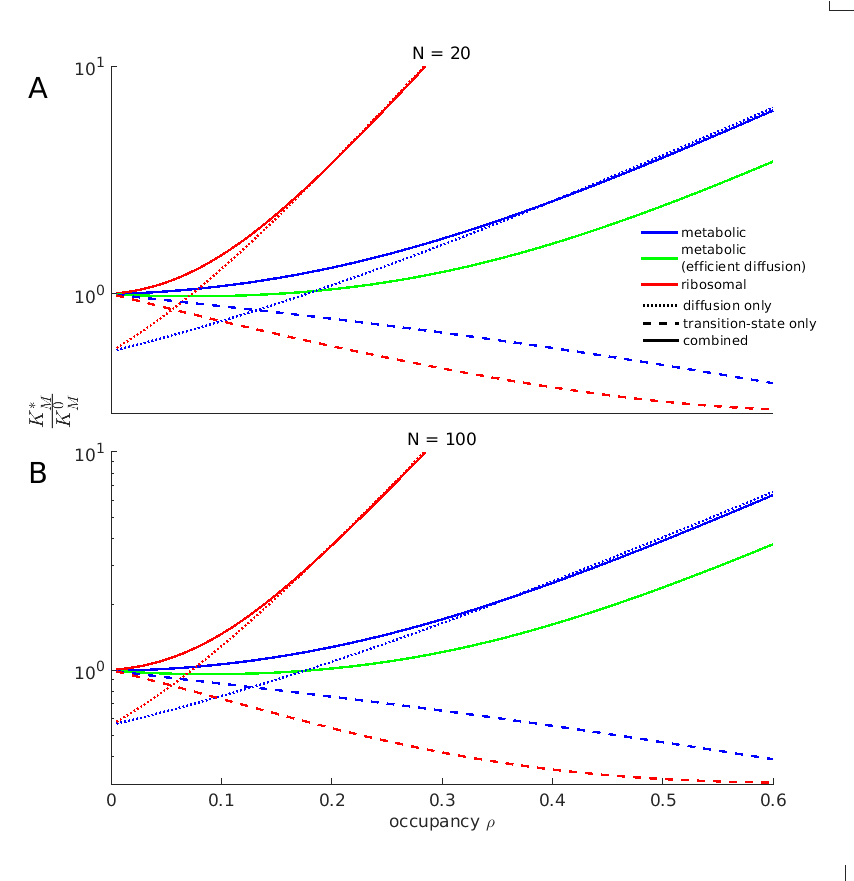


**Supplementary Figure S7. Opposing effects of molecular crowding on the Michaelis parameter** $K_{\text{M}}^{*}/K_{\text{M}}^{0}$ **through perturbations of diffusion and of Gibbs free energies.** Simulations considered the individual and combined effects of diffusion limitation and transition state limitation for a parallel system of ribosomal reactions (red) and for a linear system of metabolic reactions (blue: *θ*=2.3; green: combined effect with a stronger bias towards transition state limitation, *θ*=4.6). **(A)** *N*=20 and **(B)** *N*=100.
