# Supplementary material for: Optimal density of bacterial cells
Source: PLoS Comput Biol. 2023 Jun 12;19(6):e1011177. doi: 10.1371/journal.pcbi.1011177 (PMC10289677; doi:10.1371/journal.pcbi.1011177)
Supplement: S8 Fig — Symbols on the x-axis labels are; MM: minimal medium; man: mannose; gly: glycerol; glu: glucose; CAA: casamino acids; RDM: rich dry medium. To limit the y-axis range, both panels do not show the outlier points. Each bar in (A) corresponds to the distribution of ρDM measurements of wildtype E. coli cells (MG1655) cultured in the same nutritional condition, reported in Oldewurtel et al. 2021. With the exception of MM+gly, all conditions of minimal media have indistinguishable mean cytosolic mass density (See S1A Table for the results of the tests). Each bar in (B) corresponds to the ρ estimated from the ρDM measurements of the same condition using Eq. (12a) and Eq. (12b). r¸ the RNA/protein mass ratio, is necessary in the calculation of ρ; we estimated r from the growth rate μ using the MATLAB interpolation function ‘interp1’ and the μ-r measurements of wildtype E. coli in S3 Table of Dai et al. 2016. In contrast to (A), all distributions here have different mean values (See S1B Table for the results of the statistical tests). Excluding MM+gly, this graph shows a trend of decreasing ρ with increasing μ. (DOCX) [file pcbi.1011177.s008.docx]

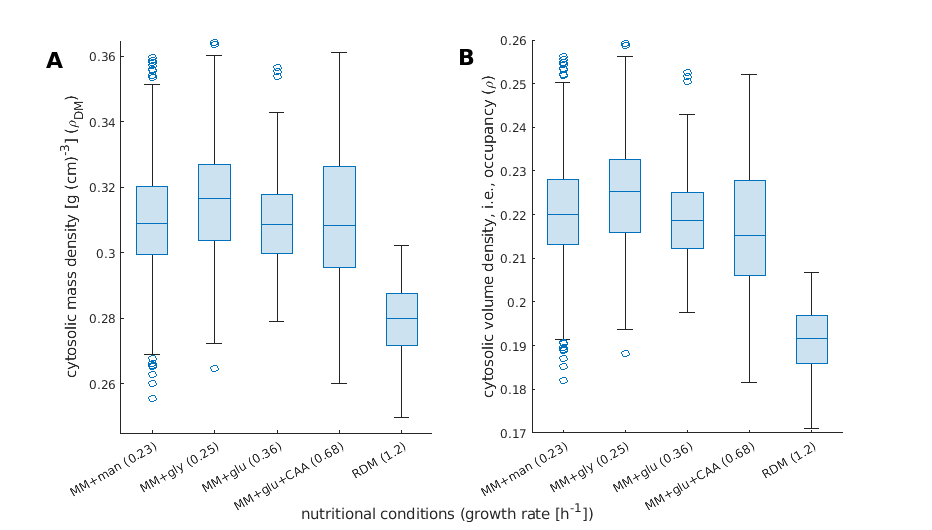


**Supplementary Figure S8.** Boxplot displaying the distribution of **(A)** cytosolic mass density (*ρ*_DM_) and **(B)** occupancy (*ρ*) across different nutritional environments. Symbols on the x-axis labels are; MM: minimal medium; man: mannose; gly: glycerol; glu: glucose; CAA: casamino acids; RDM: rich dry medium. To limit the y-axis range, both panels do not show the outlier points. Each bar in **(A)** corresponds to the distribution of *ρ*_DM_ measurements of wildtype *E. coli* cells (MG1655) cultured in the same nutritional condition, reported in Oldewurtel et al. [1]. With the exception of MM+gly, all conditions of minimal media have indistinguishable mean cytosolic mass density (See **Table S1A** for the results of the tests). Each bar in **(B)** corresponds to the *ρ* estimated from the *ρ*_DM_ measurements of the same condition using Eq. (12a) and Eq. (12b). *r*¸ the RNA/protein mass ratio, is necessary in the calculation of *ρ*; we estimated *r* from the growth rate *µ* using the MATLAB interpolation function ‘interp1’ and the *µ*-*r* measurements of wildtype *E. coli* in Suppl. Table 3 of Dai et al. [2]. In contrast to **(A)**, all distributions here have different mean values (See **Table S1B** for the results of the statistical tests). Excluding MM+gly, this graph shows a trend of decreasing *ρ* with increasing *µ*.

# References

1. Oldewurtel, E. R., Kitahara, Y., & van Teeffelen, S. (2021). Robust surface-to-mass coupling and turgor-dependent cell width determine bacterial dry-mass density. In Proceedings of the National Academy of Sciences (Vol. 118, Issue 32). Proceedings of the National Academy of Sciences. <https://doi.org/10.1073/pnas.2021416118>
2. Dai, X., Zhu, M., Warren, M., Balakrishnan, R., Patsalo, V., Okano, H., Williamson, J. R., Fredrick, K., Wang, Y.-P., & Hwa, T. (2016). Reduction of translating ribosomes enables Escherichia coli to maintain elongation rates during slow growth. In Nature Microbiology (Vol. 2, Issue 2). Springer Science and Business Media LLC. <https://doi.org/10.1038/nmicrobiol.2016.231>
