# Supplementary material for: Optimal density of bacterial cells
Source: PLoS Comput Biol. 2023 Jun 12;19(6):e1011177. doi: 10.1371/journal.pcbi.1011177 (PMC10289677; doi:10.1371/journal.pcbi.1011177)
Supplement: S1 Table — ρ is calculated from ρDM and r, the RNA/protein mass ratio, using Eq. (12a) and Eq. (12b). r is estimated from the μ using the MATLAB interpolation function ‘interp1’ and the μ-r measurements of wildtype E. coli reported in S3 Table of Dai et al. 2016. Each cell in the two tables corresponds to the p-value of the Wilcoxon rank-sum test, colored with red (significantly different / dissimilar) and blue (not significantly different / similar) with cutoff significance level 0.05. Symbols of the condition labels are; MM: minimal medium; man: mannose; gly: glycerol; glu: glucose; CAA: casamino acids; RDM: rich dry medium. S1A Table. P-values of spearman correlation to compare the cytosolic dry mass density ρDM between different nutritional conditions. S1B Table. P-values of spearman correlation to compare the cytosolic occupancy ρ between different nutritional conditions. (DOCX) [file pcbi.1011177.s009.docx]

**Supplementary Table S1.** Pairwise comparisons, based on two-sided Wilcoxon rank-sum tests, of cytosolic mass density *ρ*_DM_ (**A**) and cytosolic occupancy *ρ* (**B**) of wildtype *E. coli* (MG1655) cells cultured in different nutritional conditions. *ρ* is calculated from *ρ*_DM_ and *r*, the RNA/protein mass ratio, using Eq. (12a) and Eq. (12b). *r* is estimated from the *µ* using the MATLAB interpolation function ‘interp1’ and the *µ*-*r* measurements of wildtype *E. coli* reported in Supp Table 3 of Dai et al. [1]. Each cell in the two tables corresponds to the p-value of the Wilcoxon rank-sum test, colored with red (significantly different / dissimilar) and blue (not significantly different / similar) with cutoff significance level 0.05. Symbols of the condition labels are; MM: minimal medium; man: mannose; gly: glycerol; glu: glucose; CAA: casamino acids; RDM: rich dry medium.

**Supplementary Table S1A.** P-values of spearman correlation to compare the cytosolic dry mass density *ρ*_DM_ between different nutritional conditions.

| *ρ*_DM_ | MM+gly | MM+glu | MM+glu+AA | RDM |
| --- | --- | --- | --- | --- |
| MM+man | 4.79E-10 | 0.59409 | 0.67293 | 3.63E-50 |
| MM+gly |  | 2.05E-07 | 4.81E-05 | 1.24E-49 |
| MM+glu |  |  | 0.96644 | 7.49E-44 |
| MM+glu+AA |  |  |  | 4.40E-34 |

**Supplementary Table S1B.** P-values of spearman correlation to compare the cytosolic occupancy *ρ* between different nutritional conditions.

| *ρ* | MM+gly | MM+glu | MM+glu+AA | RDM |
| --- | --- | --- | --- | --- |
| MM+man | 7.90E-09 | 0.020149 | 3.14E-07 | 8.38E-59 |
| MM+gly |  | 3.95E-10 | 1.69E-14 | 6.48E-55 |
| MM+glu |  |  | 0.0045057 | 2.86E-48 |
| MM+glu+AA |  |  |  | 2.41E-40 |

# References

1. Dai, X., Zhu, M., Warren, M., Balakrishnan, R., Patsalo, V., Okano, H., Williamson, J. R., Fredrick, K., Wang, Y.-P., & Hwa, T. (2016). Reduction of translating ribosomes enables Escherichia coli to maintain elongation rates during slow growth. In Nature Microbiology (Vol. 2, Issue 2). Springer Science and Business Media LLC. <https://doi.org/10.1038/nmicrobiol.2016.231>
