# Supplementary material for: Optimal density of bacterial cells
Source: PLoS Comput Biol. 2023 Jun 12;19(6):e1011177. doi: 10.1371/journal.pcbi.1011177 (PMC10289677; doi:10.1371/journal.pcbi.1011177)
Supplement: S2 Table — (DOCX) [file pcbi.1011177.s010.docx]

**Supplementary Table S2.** List of metabolites for the glucose only nutritional environment; it is a reproduction of Table S3 in page 97 of Ref [1], excluding the 20 amino acids.

| Metabolite | Exchange reaction in sybilccFBA | Value^1^ |
| --- | --- | --- |
| D-Glucose | R_EX_glc_e__b | -1000 |
| Potassium + | R_EX_k_e__b | -1000 |
| Thiamin | R_EX_thm_e__b | -1000 |
| Sodium | R_EX_na1_e__b | -1000 |
| Calcium | R_EX_ca2_e__b | -1000 |
| Ammonia | R_EX_nh4_e__b | -1000 |
| Magnesium | R_EX_mg2_e__b | -1000 |
| Iron 3+ | R_EX_fe3_e__b | -1000 |
| Zinc | R_EX_zn2_e__b | -1000 |
| Copper 2+ | R_EX_cu2_e__b | -1000 |
| Manganese 2+ | R_EX_mn2_e__b | -1000 |
| Cobalt 2+ | R_EX_cobalt2_e__b | -1000 |
| Chloride | R_EX_cl_e__b | -1000 |
| Carbon dioxide | R_EX_co2_e__b | -1000 |
| H+ | R_EX_h_e__b | -1000 |
| Water | R_EX_h2o_e__b | -1000 |
| Sulphate | R_EX_so4_e__b | -1000 |
| Phosphate | R_EX_pi_e__b | -1000 |
| Oxygen | R_EX_o2_e__b | -1000 |
| Molybdate | R_EX_mobd_e__b | -1000 |
| Nickel 2+ | R_EX_ni2_e__b | -1000 |

^1^ values in mM (g dry weight)^-1^ h^-1^

# References

1. Alzoubi, D. (2019). Pleiotropy and Epistasis in constraint-based models of microbial metabolism. Dissertation. Heinrich Heine University Düsseldorf.
