# Supplementary material for: Optimal density of bacterial cells
Source: PLoS Comput Biol. 2023 Jun 12;19(6):e1011177. doi: 10.1371/journal.pcbi.1011177 (PMC10289677; doi:10.1371/journal.pcbi.1011177)
Supplement: S3 Table — (DOCX) [file pcbi.1011177.s011.docx]

**Supplementary Table S3.** The nutrients in the environment for the glucose + 20 amino acids medium includes those listed in Supplementary Table S2, plus the following 20 amino acids.

| Metabolite | Exchange reaction in sybilccFBA | Value^1^ |
| --- | --- | --- |
| … ... [Metabolites in Supplementary Table S2] … ... | | |
| L-Alanine | R_EX_ala_L_e__b | -1000 |
| L-Arginine | R_EX_arg_L_e__b | -1000 |
| L-Asparagine | R_EX_asn_L_e__b | -1000 |
| L-Aspartate | R_EX_asp_L_e__b | -1000 |
| L-Cysteine | R_EX_cys_L_e__b | -1000 |
| L-Glutamine | R_EX_gln_L_e__b | -1000 |
| L-Glutamate | R_EX_glu_L_e_ | -1000 |
| Glycine | R_EX_gly_e__b | -1000 |
| L-Histidine | R_EX_his_L_e__b | -1000 |
| L-Isoleucine | R_EX_ile_L_e__b | -1000 |
| L-Leucine | R_EX_leu_L_e__b | -1000 |
| L-Lysine | R_EX_lys_L_e__b | -1000 |
| L-Methionine | R_EX_met_L_e__b | -1000 |
| L-Phenylalanine | R_EX_phe_L_e__b | -1000 |
| L-Proline | R_EX_pro_L_e__b | -1000 |
| L-Serine | R_EX_ser_L_e__b | -1000 |
| L-Threonine | R_EX_thr_L_e__b | -1000 |
| L-Tryptophan | R_EX_trp_L_e__b | -1000 |
| L-Tyrosine | R_EX_tyr_L_e__b | -1000 |
| L-Valine | R_EX_val_L_e__b | -1000 |

^1^ values in mM (g dry weight)^-1^ h^-1^
