# Supplementary material for: Optimal density of bacterial cells
Source: PLoS Comput Biol. 2023 Jun 12;19(6):e1011177. doi: 10.1371/journal.pcbi.1011177 (PMC10289677; doi:10.1371/journal.pcbi.1011177)
Supplement: S4 Table — (DOCX) [file pcbi.1011177.s012.docx]

**Supplementary Table S4.** The list of nutrients in the environment of the rich medium; it is a reproduction of Table S4 in page 98 of Ref [1], excluding biotin, pyridoxine, selenate and selenite as their exchange reactions are missing in the iAF1260 model.

| Metabolite | Exchange reaction in sybilccFBA | Value^1^ |
| --- | --- | --- |
| Cob(I)alamin | R_EX_cbl1_e__b | -0.01 |
| Adenine | R_EX_ade_e__b | -1000 |
| D-Alanine | R_EX_ala_D_e__b | -1000 |
| L-Alanine | R_EX_ala_L_e__b | -1000 |
| L-Arginine | R_EX_arg_L_e__b | -1000 |
| L-Asparagine | R_EX_asn_L_e__b | -1000 |
| L-Aspartate | R_EX_asp_L_e__b | -1000 |
| Cytosine | R_EX_csn_e__b | -1000 |
| L-Cysteine | R_EX_cys_L_e__b | -1000 |
| D-Cysteine | R_EX_cys_D_e__b | -1000 |
| L-Glutamine | R_EX_gln_L_e__b | -1000 |
| L-Glutamate | R_EX_glu_L_e_ | -1000 |
| Glycine | R_EX_gly_e__b | -1000 |
| Guanine | R_EX_gua_e__b | -1000 |
| L-Histidine | R_EX_his_L_e__b | -1000 |
| L-Homoserine | R_EX_hom_L_e__b | -1000 |
| L-Isoleucine | R_EX_ile_L_e__b | -1000 |
| L-Leucine | R_EX_leu_L_e__b | -1000 |
| L-Lysine | R_EX_lys_L_e__b | -1000 |
| L-Methionine | R_EX_met_L_e__b | -1000 |
| Nicotinamide  mononucleotide | R_EX_nmn_e__b | -1000 |
| L-Phenylalanine | R_EX_phe_L_e__b | -1000 |
| L-Proline | R_EX_pro_L_e__b | -1000 |
| D-Serine | R_EX_ser_D_e__b | -1000 |
| L-Serine | R_EX_ser_L_e__b | -1000 |
| Thiamin | R_EX_thm_e__b | -1000 |
| L-Threonine | R_EX_thr_L_e__b | -1000 |
| Thymine | R_EX_thym_e__b | -1000 |
| L-Tryptophan | R_EX_trp_L_e__b | -1000 |
| L-Tyrosine | R_EX_tyr_L_e__b | -1000 |
| Uracil | R_EX_ura_e__b | -1000 |
| L-Valine | R_EX_val_L_e__b | -1000 |
| Glucose | R_EX_glc_e__b | -1000 |
| H+ | R_EX_h_e__b | -1000 |
| Water | R_EX_h2o_e__b | -1000 |
| Calcium | R_EX_ca2_e__b | -1000 |
| Chloride | R_EX_cl_e__b | -1000 |
| Carbon dioxide | R_EX_co2_e__b | -1000 |
| Cobalt 2+ | R_EX_cobalt2_e__b | -1000 |
| Copper 2+ | R_EX_cu2_e__b | -1000 |
| Iron 2+ | R_EX_fe2_e__b | -1000 |
| Iron 3+ | R_EX_fe3_e__b | -1000 |
| Potassium+ | R_EX_k_e__b | -1000 |
| Magnesium | R_EX_mg2_e__b | -1000 |
| Manganese 2+ | R_EX_mn2_e__b | -1000 |
| Molybdate | R_EX_mobd_e__b | -1000 |
| Sodium | R_EX_na1_e__b | -1000 |
| Ammonia | R_EX_nh4_e__b | -1000 |
| Nickel 2+ | R_EX_ni2_e__b | -1000 |
| Oxygen | R_EX_o2_e__b | -1000 |
| Phosphate | R_EX_pi_e__b | -1000 |
| Sulphate | R_EX_so4_e__b | -1000 |
| Tungstate | R_EX_tungs_e__b | -1000 |
| Zinc | R_EX_zn2_e__b | -1000 |

^1^ values in mM (g dry weight)^-1^ h^-1^

# References

1. Alzoubi, D. (2019). Pleiotropy and Epistasis in constraint-based models of microbial metabolism. Dissertation. Heinrich Heine University Düsseldorf.
