# Supplementary material for: Optimal density of bacterial cells
Source: PLoS Comput Biol. 2023 Jun 12;19(6):e1011177. doi: 10.1371/journal.pcbi.1011177 (PMC10289677; doi:10.1371/journal.pcbi.1011177)
Supplement: Attachment — Submitted filename: Response to Reviewers.docx [file pcbi.1011177.s013.docx]

## Reviewer #1

**Major criticism:**

***1)The only comparison with experimental data made by the authors about the growth rate dependence of the bacterial cytosol density is with the data from Oldewurtel et al.. The paper measured the dry mass of bacterial cells at the single-cell level. As the authors also wrote in the discussion, the data have about a 5% of deviation. The authors state the data supports a 6% of reduction of the concentration between the very low growth rate (close to zero) to 0.7/hour, but it is not a meaningful difference with the given spread of data. In addition, the authors use the linear extrapolation of the ribosome fraction from Scott et al. [1] in this calculation. However, the data in the paper by Dai et al. that the authors also cite in [34] show a clear deviation from the linear fit in this range of growth rate. If the data in Dai et al. is used, the predicted difference will be even smaller even if one follows the authors' calculation method, making the prediction less relevant compared to the measurement precision. For a higher growth rate, the decrease is claimed to be 13% from the data and the model prediction is 15%. The authors only state that they are consistent and that I agree that they are not inconsistent, but I don’t see them as an agreement to support the theory.***

**Response:** The reviewer pointed out that the cytosolic mass density measurements in Oldewurtel et al. have a large spread, so it is difficult to determine whether the cytosolic mass density at higher growth rates is indeed lower than that at lower growth rates. To address this concern, we have performed further statistical analyses. Oldewurtel et al. measured the cytosolic mass density along 10 different wildtype *E. coli* (MG1655) cell growth trajectories (5 different nutritional conditions, each with 2 samples). The new **Fig. S8** shows boxplots that summarize the data for each of the nutritional conditions. We applied Wilcoxon rank-sum tests to determine whether the mass densities between these distributions was statistically significantly different (**Table S1a**). With the exception of one outlier (growth on minimal medium with glycerol), the mass density distributions for the four minimal media have means that are indistinguishable (Fig. S8a). We then converted the mass densities to volume densities (Fig. S8b). Now, we found statistically significant differences between all conditions that followed the predicted trend: volume density decreases with increasing growth rate (again with the exception of the glycerol condition).

Further, the reviewer also criticized that the linear approximation of Scott et al. to predict the RNA/protein mass ratio (r) from growth rate is inaccurate, because the measurements from Dai et al. show a systematic deviation from linearity. In response, we repeated all the relevant calculations, using Dai et al.’s data instead of Scott et al.’s linear approximation. Using Scott et al.’s approximation, we concluded that cytosolic volume density drops by 13%.; using Dai et al.’s, we found that the cytosolic volume density drops by 12%.

Thus, we maintain that our predictions—a 7% decrease between slow and fast growth on minimal media, and a 15% decrease between fast growth on minimal media and ultrafast growth on rich medium – are not only consistent with the observed reductions—4% and 11%, respectively—but that this also supports our hypothesis.

**Action:** As suggested by the reviewer, we changed the conversion factor between mass density and occupancy from Scott et al.’s linear model to an interpolation between the factors observed by Dai et al. (changes made in the Method section “Conversion of experimental dry mass density to occupancy”). We added the boxplots for the experimental mass density distributions (Suppl. Fig. S8a) and the corresponding occupancies (Suppl. Fig. S8b), and performed statistical tests to compare the mean mass densities (Suppl. Table 1a) and the mean occupancies (Suppl. Table 1b).

**2a) There is a large body of research on how cell size is regulated in balanced growth in E. coli. Since the cell size (volume) and the content together give the density, I found it rather misleading that the current manuscript does not talk about the cell size volume at all. For example, the paper [6] states in the significance statement that ” First, cells expand surface area, rather than volume, in proportion with biomass growth. Second, cell width is controlled independently, with an important influence of turgor pressure.” These regulations then result in a certain dry mass density for a given growth rate. How is this related to the current paper’s point of view?**

**Response:** We agree with the reviewer that it is desirable to include a short description of current understanding on how an *E. coli* cell maintains its density based on regulation of cell surface area and volume, and how this is related to our work.

**Action:** We have added a corresponding paragraph to the Introduction (line 67-77).

**2b) Another body of literature that the current manuscript is not discussing at all is the coupling between cell size and DNA replication, reviewed e.g. in Jun et al. Rep. Prog. Phys. 81 (2018) 056601. In this view, the cell size is determined by the initiation mass per origin of replication. This does not directly tell the concentration of various macromolecules in the cell, but clearly indicates that the packing of DNA is a very important determinant to determine the volume of the cell. To me, it sounds unlikely that DNA replication plays no role in determining the density of molecules in the cell. For example, I could imagine a scenario where the cell volume needs to increase quite a lot for faster growth to accommodate the space for DNA replication, but making all the proteins at the same proportionality requires a quite large increase in the production rate of the proteins, that cell cannot catch up and density decrease with the growth rate - in other words, the decrease of the density is a side effect.**

**Response:** This is an important point, and we thank the reviewer for bringing it to our attention. Indeed, a high number of replication rounds at a higher growth rate may also contribute to the lower cell density observed.

**Action:**  We included a paragraph in the Discussion to explain this alternative mechanism (line 413-419).

**2c) The current manuscript does not discuss any argument against such possibilities. Also, while there may be limited literature that determined dry mass at the single cell level, there are many pieces of literature that have average numbers for macromolecules for various growth conditions (e.g. Bremer and Dennis), and that is all the authors need to compare their theory with, which is only about the mean values. Listing and discussing various data from the literature on growth rate dependence of the cell volume and macromolecule mass and giving a critical evaluation of their own proposal is a necessity when proposing a speculative idea.**

**Response:** While there is data on average numbers of macromolecules, this data is typically estimated per cell, per optical density, or per dry mass density. Unfortunately, there is no straightforward way to convert these measurements into volume occupancies. However, there are indeed earlier experiments based on cell buoyant density measurements (done with a density gradient in centrifugation) and also based on cellular dry mass and volume inferred from optical absorption. These experiments indicated that the *E. coli* cellular density is approximately constant across growth conditions, a conclusion that was confirmed by the more accurate recent experiments by Sven van Teeffelen’s group. However, the latter study found a statistically significant density decrease of around 10% at the fastest growth rates (see our response to comment 1).

**Action:** We included a brief description of previous cell density measurements before in the Introduction section of the manuscript (line 57-64).

**A technical comment**

**Line 635 gives the translation rate in the model, and the maximum translation rate k_cat^R is set to 22.0/s. But this number is the measured ribosomal elongation rate, which is already affected by molecular crowding effects. Shouldn’t it be set so that the overall rate becomes consistent with this number after one puts in the crowding and the substrate concentration in the entire expression?**

**Response:** In our formulation, molecular crowding affects *K*_M_ but not *k*_cat_. Therefore, we can simply use the empirical value of *k*_cat_^R^ without worrying about the effect of crowding.

**Action:** none.

## Reviewer #2

**1) In the first part of the methods, the crowding-adjusted Michaelis parameter K_m^* is derived, however, the derivation is based on K_m ~ k_off/k_on (and later equality is used) rather than the conventional definition of K_m = (k_off+k_cat)/k_on. The change of definition is not commented on but should have effects on the overall derivation. Please can the authors comment on this choice of definition and its impact on the results?**

**Response:** Indeed, our definition of K_M_ (k_off_/k_on_) (A) differs from the conventional definition of (k_off_+k_cat_)/k_on_ (B). Definition A assumes that k_off_ >> k_cat_, and is used generally when considering complex reactions (Liebermeister & Klipp, 2006).

Definition B is applicable only when there is a single substrate in the enzymatic reaction. However, many metabolic reactions have more than one substrate, and definition A is more convenient in those cases. Let us consider an example with a metabolic reaction having 2 substrates, S_1_ and S_2_. The enzyme can form a duplet complex by either binding with S_1_ (S_1_-E) or S_2_ (E-S_2_), but the reaction only takes place when they form a triplet (S_1_-E-S_2_). In this case, we need to consider the different states of the enzyme (free, bound to S_1_, bound to S_2_, bound to both) to calculate the reaction rate, which can be written as

$${v=k}_{cat}[E]\frac{[S_{1}][S_{2}]/(K_{M1}K_{M2})}{1+[S_{1}]/K_{M1}+[S_{2}]/K_{M2}+[S_{1}][S_{2}]/(K_{M1}K_{M2})}$$

Here, both K_M_s are based on definition A, i.e., K_M1_ = (k_off1_/k_on1_) and K_M2_ = (k_off2_/k_on2_). k_on1_ and k_on2_ are the rates for S_1_ and S_2_ to bind with E, respectively. k_off1_ and k_off2_ are the rates for the substrate to dissociate from the complex S_1_-E and E-S_2_. See Liebermeister and Klipp (2006) for further explanations.

Definition A allows us to more easily separate an enzymatic reaction into a 2-step process (complex formation and complex proceeding to make the product). For this reason, Minton (1998) derived the formulations using definition A. We followed this previous work in using definition A.

References:

- Liebermeister, W., & Klipp, E. (2006). Bringing metabolic networks to life: convenience rate law and thermodynamic constraints. In Theoretical Biology and Medical Modelling (Vol. 3, Issue 1). Springer Science and Business Media LLC. <https://doi.org/10.1186/1742-4682-3-41>
- Minton, A. P. (1998). [7] Molecular crowding: Analysis of effects of high concentrations of inert cosolutes on biochemical equilibria and rates in terms of volume exclusion. In Methods in Enzymology (pp. 127–149). Elsevier. <https://doi.org/10.1016/s0076-6879(98)95038-8>

**Action:** We now briefly explain the difference between definitions A and B, and the argument for using A, in the Methods (line 448-457).

**2) I wonder to what extent the results could be strengthened via an analytical derivation of the optimal cytosolic occupancy. There is mention of an iterative procedure due to the interdependency of [ES] and K_m^*, but the algorithmic details are not specified and it is unclear to me whether at least for the linear and parallel models analytical results may be feasible.**

**Response:** We have actually tried to solve the problem analytically, but were unable to find an analytical form. That’s why we solve the model numerically in the manuscript. We regret that we did not provide enough information on the algorithmic details.

**Action:** We have extended the description of the iterative procedure to clarify algorithmic details (line 572-580).

**3a) After eq (2) it is mentioned that transition-state limit dominates when \theta -> \inf, but it is not obvious to see in eq (2) how this would result in K_M^*=K_M^0. After more careful reading of the methods section I understood that in the ideal ground case also \Gamma=1, which then gives equality. This could be presented more clearly.**

**Response:** We thank the reviewer for pointing out the ambiguity in the manuscript near Eq (2).

**Action:** We tried to remove the ambiguity by including the information that was outlined in the reviewer’s comment (line 166-173).

**3b) Figures could be presented more concisely. For example, there seems to be redundancy between Fig 5 (which also doesn’t have an explanatory caption) and Fig S2.**

**Response:** Fig. 5 shows the optimal density for the main whole cell model at different values of N (number of enzymes) and s_ext_ (nutrient concentration). Fig. S2 shows the optimal density for both the main model (replicating the information in Fig. 5) and a modified whole cell model. The focus of Fig. 5 is the main model, whereas the focus of Fig. S2 is the modified model, with the information from Fig. 5 repeated to facilitate an easy comparison. To not distract the readers in the main text, we believe that it is better not to combine the two plots.

**Action:** To emphasize the focus of Fig. S2, we changed the line styles. In addition, we have edited the captions of both Fig. 5 and Fig. S2 to explain better what is being shown.

**In Fig 7, panels are not labelled, and the figure also looks more appropriate for the SI (to support Fig 6).**

**Action:** As suggested, we moved Fig. 7 to the SI and added labels to improve the presentation.

**Overall, the content of figures is presented very generously regarding space on the page, the authors may want to consider smaller plots and perhaps using insets (e.g. combining Figs 4 & 5).**

**Response:** The minimal width for figures in PLOS journals is 2.63 inch, roughly a third of the width of an A4 paper. We believe that this minimal width is fitting for Fig. 4 and Fig. 5, and so we prefer to keep the layout of the figures.

**Action:** None.

**4) I feel an overview schematic, illustrating the effects of the parameters \Gamma, \rho and \theta, could improve clarity of presentation**

**Response:** We agree with the reviewer that it would be desirable to include a schematic illustrating the effects of the three parameters of the crowding model, Γ, ρ, and θ. However, we have not found a good way to represent Γ and its effects. θ describes the balance between the effects of diffusion limitation and transition state limitation, and is hence closely related to Fig. 1. Since ρ is simply the volume density, we believe that an extra figure describing ρ – such as drawing two schematic cells with different densities of molecules – is not going to be very informative.

**Action:** We now include θ in Fig. 1 to illustrate its effect.

**5) I would suggest a consistent use of math fonts to clearly indicate variables in the main text.**

**Response:** As noted by the reviewer, there is indeed an inconsistent layout of the mathematics. We apologize for the resulting ambiguity, which was caused by an incomplete switch between different text editing systems.

**Action:** We now use the same formatting for all symbols and equations throughout the manuscript.
