# Supplementary material for: Optimal density of bacterial cells
Source: PLoS Comput Biol. 2023 Jun 12;19(6):e1011177. doi: 10.1371/journal.pcbi.1011177 (PMC10289677; doi:10.1371/journal.pcbi.1011177)
Supplement: Attachment — Submitted filename: reply_letter.docx [file pcbi.1011177.s014.docx]

# Reviewer #1

*1. The authors have responded to most of the reviewers' criticism. Discussions on other possible mechanisms and their relation to other literature are included in the updated manuscript. Most importantly, the authors performed a more thorough analysis in comparison with the experimental data from ref. 10, and the claim is toned down that the current analysis is consistent with those data, but not necessarily the only explanation of the observation.*

*With the new analysis regarding data with ref. 10, I still have a concern as below and I strongly hope that the authors add a discussion on this. If that is done in a convincing manner, I believe this paper is worth publishing to raise the discussion of this interesting claim in the community.*

**Response:** We thank the reviewer for this positive assessment of our revisions.

*2. What I am concerned about by looking at the newly added statistical analysis in Table S1A is that it shows the \rho_DM for MM+gly medium is significantly different from any other growth condition and the authors ignore it in the analysis in the main text. In fact, I agree intuitively with the authors that the data feels that only RDM is truly different (they also have extremely low p-value). But if we accept only the extremely low p-value only, then I would say Table S1B is showing that only RDM is different for \rho and not other conditions. Of course, one should not tune the p-value threshold after performing the analysis. Just it feels somewhat strange that these features are ignored in the main text.*

**Response:** We agree that it is noteworthy that the mean observed mass density in growth on MM + gly is statistically significantly higher than in other minimal media conditions. However, the mass density distributions across all minimal media – including MM + gly – overlap strongly, indicating that the difference may not be physiologically important. In contrast, the observed mass density distributions differ markedly between all minimal media and RDM, emphasized also by the much smaller *p*-values.

**Action:** We now briefly discuss these issues in lines 353-357.

*3. In addition, my feeling is that the real source of these somewhat inconclusive analyses is the fact that the data have a large variance. This gives me another concern that even if the median may be significantly different for \rho or \rho_DM, the real cells do not seem to be really optimized to that value - in other words, the cells are perfectly fine to deviate from the value of \rho individually from the predicted optimum. If there is selection pressure to converge to the predicted optimum, shouldn’t that also select for reducing the variance, too? How much benefit in the growth rate does the population get, if there is such a wide deviation?*

*Overall, discussing the statistical significance of the median does not solve my concern in terms of the experimental evidence. It will be desirable if the authors can thoroughly discuss how the authors' hypothesis explains the variance of the data.*

**Response:** There is indeed a strong variability of mass density across cells grown in the same conditions, which amounts to a coefficient of variation of around 5%. While our model simulations indicate that deviations from the optimal \rho of this magnitude come with fitness penalties of only around 1e-4, we would still expect them to be under negative selection, given bacterial population sizes N_e >> 1e8. Thus, while our deterministic model predicts some variability, it does not predict variation of this magnitude. These considerations indicate that additional factors beyond selection for fast growth modify the density of individual cells either actively – through regulation – or passively – through the coupling of mass density to other factors. However, these observations do not invalidate our model, but rather indicate that other factors can be seen as perturbations of the state optimal for rapid growth, because our results are consistent with the average densities of cells across different growth conditions.

**Action:** We have added an extra paragraph in the Discussion to explain this notion (lines 417-427).

# Reviewer #2

*My concerns have been satisfactorily addressed.*

**Response:** We thank the reviewer for this positive assessment of our revisions.
